# Dental Education

**Published:** 1880-02

**Authors:** K. B. Davis

**Affiliations:** Springfield


### DENTAL EDUCATION.

BY K. B. DAVIS, SPRINGFIELD.

Considered in all its relations, there is no more important subject to the dental profession than Dental Education. It has been, and doubtless will continue to be, a very prolific theme for discussion, both in our societies and journals. As the discussion of this subject progresses, a deeper interest seems to manifest itself in our profession in behalf of a more thorough education for all who wish to enter its ranks.

It will doubtless be admitted by all who wish to promote the best interests of the profession, that no one who now wishes to enter the ranks of dental surgery, should do so, only after receiving a thorough dental education. Both in order to make this special training as proficient as it should be, some preliminary education is an essential requisite. What, then, should this be? It should, evidently be more than has heretofore been required of those entering the profession. It is certainly obvious to the careless observer, that a very large proportion of those now practicing dentistry, are sadly deficient in preliminary education. They can barely read and write, but can not correctly spell many of the shortest words in our language. This deplorable condition is not only true of dentistry, but also of medicine.

The English Parliament has lately passed an act, that dentists shall hereafter possess a good preliminary education, before they begin their professional training. Under this law, the prescribed examination covers English, Latin, arithmetic, algebra, geometry, geography, English history, and any one of the following subjects, at the the candidate's option: Greek, French, German, mechanics, chemistry, botany, and zoology. To attempt the establishment of such a standard of preliminary education in our country, would, perhaps, at the present time, be impossible; still, no argument is needed to prove that such conditions of entrance as these would, in a few years, greatly improve the mass of the profession. If our profession in this country is to maintain and advance its rank in the scientific world, it *must be defended* by similar requisitions against the incursion of uneducated men.

The profession should, undoubtedly, establish some standard for the preliminary education of its members, and this should, under no circumstances, be less than a good English education. But in the selection of students, let us always endeavor to secure those with a scientific or classical education. So long as dentists are as careless in the selection of their students, as they have been hitherto, so long will our dental colleges have trouble in elevating the standard of dental education. There is too little discrimination by dentists in the selection of students.

Our colleges ask and desire the co-operation and support of the profession. But it is well for us to understand clearly, the character of the support asked for. They do not ask us to crowd the seats of their lecture-rooms with crude material, nor to send them, under cirtificate, students without any literary training, and who are barely able to make a rubber or celluloid plate, and nothing more. They do not ask us to send them young men whose ability is altogether in their fingers, but they do ask us to send them young men of high moral integrity, of good literary ability—who have the intellectual capacity to follow and properly digest a plain lecture on anatomy, chemistry, or any other subject in the college course.

The ability to eradicate these evils is in the hands of the profession, who have the training of the students. We can make the colleges just what we wish them to be. Will we perform our duties and fulfill the obligations resting upon us, as well as the colleges are doing their duties?

Now let us consider the special education of the dental student. What should this embrace? There are two prevalent views upon this subject. The one contending for a special dental course of training only; the other urging the increasing necessity for a full medical education in connection with the special dental studies, in order that the practitioner may meet every demand made upon him by the profession.

We are either artizans or medical specialists. If artizans only, we are not entitled to recognition as a branch of the medical profession; and no mechanical skill, however high, no artistic culture, however perfect, should be expected to rank for more than these. If dentistry means only mechanical and artistic ability, these can be acquired without colleges and employed without degrees; but if its claims to recognition as a specialty of medicine are worth anything, they are worth all they represent.

The question is not what dentistry has been, but what should it be; not what are the qualifications of the majority of those now practicing it, but what is its legitimate province, and what the requirements for its intelligent practice. We see no force in the assumption that, because the great mass of the profession have heretofore spent their lives in a monotonous round of purely mechanical labor, therefore they must continue to do so in future. We claim that the circle of physiological and pathological sympathies existing between the mouth and every portion of the economy, demand first a general medical education, and then special training, that the highest results in treatment may be secured. And as each year witnesses a more thorough educational training, so will the field invariably widen, until the functions of the dentist will be merged into a practice of which that of to-day is but a feeble indication.

Assuming the correctness of these conclusions, we are led to inquire what are the duties of the practitioner to the student? Or, in other words, what facilities should we afford sudents, in securing their special training. It is evidently the duty of every practitioner who assumes the responsibility of giving to the student his private training, to supply him with every aid at his command. He should, in the first place, provide him with all the latest text-books, and carefully mark out his course of reading, giving him regular reviews and examinations to ascertain what progress he is making, correcting, meanwhile, all erroneous ideas that he may fall into in relation to general principles, as well as his defects of practice. For a student should not be trained alone in the theory of dental practice, but he should be taught to manipulate in conjunction with his mental acquisitions. Nor should he, in the acquisition of his practical training, be consigned to the laboratory only, as has been too common heretofore; but he should be allowed to stand by the operating chair, and observe every detail of each operation, the perceptor giving him explicit reasons for the various processes and methods of performing both simple and difficult operations. After he has gained a sufficient amount of theoretical knowledge and ample manipulative observation at the operating chair, the student should be permitted to commence operating for such patients as he can procure, under the supervision of his preceptor, commencing first with the simple, and, as a little experience is gained, going on to more difficult operations.

Having thus spent the term of his pupilage in the energetic endeavor to make as much progress in his special studies as possible, the student is now ready to still further pursue his professional training in some one of our colleges.

At the present time, I do not think any student ought to think of commencing the practice of dentistry without securing, in addition to his private training, all the aid he can from some dental college. Therefore, I think it the imperative duty of every preceptor to urge, by every means at his command, his student, at the close of his pupilage, to attend some one of our dental schools and avail himself of every facility they afford for his further preparation to practice his chosen profession.

This brings us to a consideration of the facilities offered by our dental schools for the preparation of students for the responsible duties they have to assume in the practice of the dental profession. Are the present facilities offered to the dental student by the dental colleges, amply sufficient to qualify him to meet the requirements of the public in practice? That they offer the best means for the acquisition of professional education that we have, all must admit. But that some reforms are needed in our dental schools, all will, perhaps, as readily admit. The colleges have certainly done a great work for the profession, taking into consideration, the peculiar circumstances and difficulties by which they have been surrounded. They have had many obstacles to surmount, many experiments to try, much prejudice to combat, and withal, a sad lack of support on the part of the profession in general. It is doubtless much easier to find fault with our schools than it is to point out a correct method of eradicating the errors that seem most to mar their efficiency.

However, I think there is a disposition manifested on the part of the dental schools to reform as rapidly as circumstances will permit.

One of the most pressing needs for reform in our colleges, is to exact an admission examination of all students. No matter how ignorant, in a literary point of view, a man may be, nor whether he has had any, or what is sometimes worse than no dental pupilage, nearly, if not all of our dental schools are open to him. Until very recently, all the medical and law schools in the United States were in the same ignominious condition as regards accessibility to the ignorant; so that this disgrace is by no means peculiar to our dental schools.

Can this necessary reform in our colleges be accomplished? It undoubtedly can, if the profession is so disposed. The force of a concentrated public opinion in any profession, is the power behind the throne; it can move and accomplish almost any reform, if it is forcibly and incessantly expressed. Then, let all our colleges take this forward step in their efforts to elevate and more fully meet the requirements of the profession. Let them compel every applicant for admission to produce satisfactory evidence of not only a good moral character, but also of a good preliminary education. In doing this, they will take a most important step forward, and students thus graduated, would be rightly entitled to higher positions in the professional world than men of no literary culture or training. Then, too, would a well-defined line of demarcation be clearly drawn, which would do much towards cutting adrift and casting aside illiterate empirics, and would greatly check the malpractice of unprincipled charlatans.

The standard of qualification for graduation should be raised. In most of our colleges two terms of four months each are required for the student to complete his course. It is true, some of them have a fall and spring term of lectures. Attendance upon these terms, however, is not obligatory, so that we may say the course practically consists of two winter sessions. Can the average student become thoroughly prepared for the responsible duties of practice in so short a time? No matter how good the instruction may be, the time is certainly inadequate, and all of our schools should abolish the fragmentary fall and spring terms, and extend the regular winter course to five or six months. This would give far more time, and would not be burdensome to any young man who desires to be fully prepared to meet every responsibility resting upon him as a member of the profession.

In order to make our dental schools as efficient as they should be, they ought to be endowed. It may not be possible to bring about this desirable result under existing circumstances, but certainly our best efforts should be put forth in that direction, and sooner or later we may see it accomplished. No schools have received as little State endowment, as dental schools; one school only, so far as I know, having received any appropriation for dental education.

There is also a much needed change in the prevailing methods of examinations for graduations. A very desirable consumation would be to establish a uniform grade of teaching and scholarship in all our colleges, and then organize boards of examiners, composed of the best men in the profession, having no connection with dental schools. It would then become the duty of these boards to examine all applicants for a diploma in all our colleges. This examination should be searching, and conducted with fearless impartiality. Thus empowered, this board could establish a uniformly high grade of qualifications for graduation. Such examinations would be a test of the qualifications of students, rather than mere evidence of having passed an arbitrary and fixed term of pupilage. Then would our dental colleges strive to see how thoroughly they could prepare their students to pass this examination, and there would be no temptation to keep the standard of dental education below the public requirement. All of the examinations of these boards for degrees, should be public, at least, the questions asked should be accessible to the general profession, and the answers of candidates should likewise be open to the inspection of the profession. An oral private examination affords no guaranty, whatever, of the worth of a diploma. If we wish to make the profession of a diploma an essential preliminary to admission to practice, we will do well to insist, in the first instance, upon publicity of the examinations for the dental diploma.

May the subject of Dental Education continue to be agitated and discussed, until every member of our profession shall be forced by a strong public opinion, to go before a just and competent board of examiners and prove his fitness to practice oral surgery, and every new member that hereafter enters shall do so only after having prepared himself by attending some one of our dental schools.

